# Patient Perceptions of Artificial Intelligence in Diabetes Self-Management: Cross-Sectional Survey Study

**DOI:** 10.2196/83030

**Published:** 2026-03-16

**Authors:** Nataly Martini, Navneet Kaur Dhaliwal, Elena Alipour, Shane Scahill, Laszlo Sajtos

**Affiliations:** 1 School of Pharmacy, Faculty of Medical and Health Sciences, University of Auckland, Grafton, Auckland, 1023, New Zealand, 64 099232150; 2 Department of Marketing, Business School, University of Auckland, Auckland, New Zealand

**Keywords:** artificial intelligence, diabetes self-management, shared decision-making, patient preferences, digital health, chronic disease management

## Abstract

**Background:**

Artificial intelligence (AI) is increasingly applied in chronic disease management, including diabetes, where it has the potential to support real-time data interpretation, improve clinical decision-making, and enhance patient engagement. Although AI tools are often developed to increase efficiency and personalization, there is limited evidence on how patients perceive the role of AI in managing their condition, particularly in relation to shared decision-making (SDM) and the patient–provider relationship.

**Objective:**

This study explored how people with diabetes perceive the usefulness of AI across key self-management tasks and examined their preferences for AI versus health care provider (HCP) involvement. It also assessed predictors of AI preference and proposed a conceptual foundation for integrating AI into a triadic SDM model involving patients, HCPs, and AI.

**Methods:**

We conducted a cross-sectional online survey of adults with diabetes in New Zealand. Participants were asked to rate 7 diabetes self-management tasks in terms of (1) current HCP involvement, (2) perceived usefulness of AI, (3) comfort with HCPs using AI, and (4) preference for AI, HCP, or both in completing each task. Tasks included data collection, data interpretation, medication adherence, treatment decision-making, lifestyle management, personal reflection, and evaluation of treatment options. Both ordinary least squares regression and ordinal logistic regression (proportional odds models) were used to identify predictors of AI preference.

**Results:**

A total of 48 participants completed the survey. Of these participants, 38 (79%) were female, 27 (56%) were aged 26 to 45 years, and 26 (54%) had higher education. Mean HCP involvement across tasks was 2.82 (SD 1.23; range 1-5). AI was viewed as moderately useful overall (mean 3.67, SD 1.20), with highest usefulness for tracking (mean 4.23, SD 1.06) and interpreting information (mean 4.40, SD 0.87). Actual AI use was reported by 15/48 (31%) participants. Participants preferred HCP involvement for tasks involving treatment decision-making (17/48, 35% vs 9/48, 19%) and personal reflection (23/48, 48% vs 9/48, 19%). Across regression models, perceived usefulness of AI was a significant predictor of preference for AI in 4 tasks: data collection (*P=*.02), data interpretation (*P=*.005), treatment decision-making (*P=*.04), and lifestyle management (*P=*.046). The patient-HCP relationship significantly predicted lower preference for AI in treatment decision-making (*P=*.03) and medication adherence (ordinary least squares *P=*.005). Comfort with HCPs using AI was generally nonsignificant. Effects were modest (adjusted *R*²=0.08-0.21).

**Conclusions:**

Patients demonstrated task-specific openness to AI involvement in diabetes management, particularly for structured, data-intensive activities. These findings provide a foundation for future development and evaluation of AI-integrated SDM models. Broader exploration of technology types, relationship dynamics, and collaborative decision-making will be essential as AI becomes increasingly embedded in chronic care management.

## Introduction

### Background

Diabetes is one of the most significant global health challenges, with the number of affected adults projected to reach 783 million by 2045 [1]. In 2021, diabetes ranked among the top 10 causes of mortality worldwide, contributing to 6.7 million deaths and health care costs nearing US $1 trillion [1]. As the prevalence of diabetes continues to rise, effective care and management strategies are urgently needed. Despite advances in diabetes care, many individuals struggle with poor glycemic control, increasing their risk of complications. In New Zealand, a review of more than 3500 individuals with type 2 diabetes found that more than half had uncontrolled diabetes [2]. Contributing factors include late diagnosis, inequitable distribution of medical resources, inadequate support for lifestyle changes, and weak therapeutic relationships between patients and health care providers (HCPs) [2,3]. These factors compromise both clinical outcomes and patient engagement in care.

Shared decision-making (SDM) is a cornerstone of patient-centered care and is particularly important in chronic conditions such as diabetes, where daily self-management depends on aligning clinical guidance with the patient’s goals, values, and lived experience [4,5]. SDM fosters collaboration between patients and HCPs to select treatments and self-management strategies that reflect personal preferences and real-time health needs [6,7]. When effectively implemented, SDM builds trust, enhances confidence, and supports long-term self-efficacy [8]. However, SDM in practice is often constrained by time pressures, limited health literacy, and role confusion between patients and HCPs [9-11]. These barriers can result in mismatched expectations and leave patients to self-manage without meaningful collaboration, thereby undermining the very goals of SDM [7,12]. Furthermore, while patients value active participation in decision-making [13,14], evidence on how patients perceive SDM in their care remains scarce [15].

Artificial intelligence (AI) has emerged as a potential enabler of more responsive, patient-centered diabetes care [16]. AI-enabled technologies, including continuous glucose monitors (CGMs), mobile health apps, and smart insulin pumps, provide real-time tracking, data interpretation [3,17-21], and personalized feedback for dietary choices, exercise planning, and medication decisions [22-26]. These tools help patients manage their condition outside clinical settings, which is essential for sustained engagement and the prevention of long-term diabetes complications [23]. Recent studies also suggest AI has the potential to support aspects of SDM by simplifying complex medical information [23,26,27], helping patients to evaluate treatment options [3,28], and generating personalized insights to inform conversations with HCPs. While these capabilities align with the goals of SDM—supporting informed, values-aligned decisions—they are rarely discussed in relation to the relational dynamics that SDM entails. Existing research tends to focus on AI’s functionality in isolation, rather than its role in collaborative decision-making frameworks. Recent literature shows that patient acceptance of AI depends not only on functionality but also on trust, safety, and transparency [29,30]. Although patients may find AI tools helpful, they remain cautious about potential risks, data privacy, and the possibility of misinterpretation, and many still depend on HCP guidance to interpret AI outputs. Acceptance is further shaped by digital literacy, comorbidities, or socioeconomic factors [29].

These evolving insights highlight the need to understand not only how patients use AI tools but how they perceive AI’s role relative to HCPs in the complex, relational process of decision-making. In chronic disease contexts like diabetes, decisions such as interpreting glucose data, adjusting behaviors, or understanding treatment options are made outside clinical consultations and increasingly involve digital tools. Yet current research rarely examines how patients distribute trust, responsibility, and decision-making authority among themselves, their HCP, and AI.

This study contributes to the SDM literature in 3 ways. First, it disaggregates diabetes care into 7 distinct self-management tasks, mapping patient preferences for AI versus HCP involvement in each. Second, it empirically tests how patient-HCP, patient-AI, and HCP-AI relationships influence these preferences. Third, it proposes a novel triadic SDM framework that reimagines decision-making as a distributed process involving human and digital actors, thereby expanding current conceptualizations of SDM beyond the consultation room. The novelty of this framework lies in its examining of patients’ views on 3 dyadic interactions in SDM and in assessing their relative importance in shaping preferences for either HCPs or AI at each step of the diabetes management process.

### Conceptual Framework

**Figure 1.**
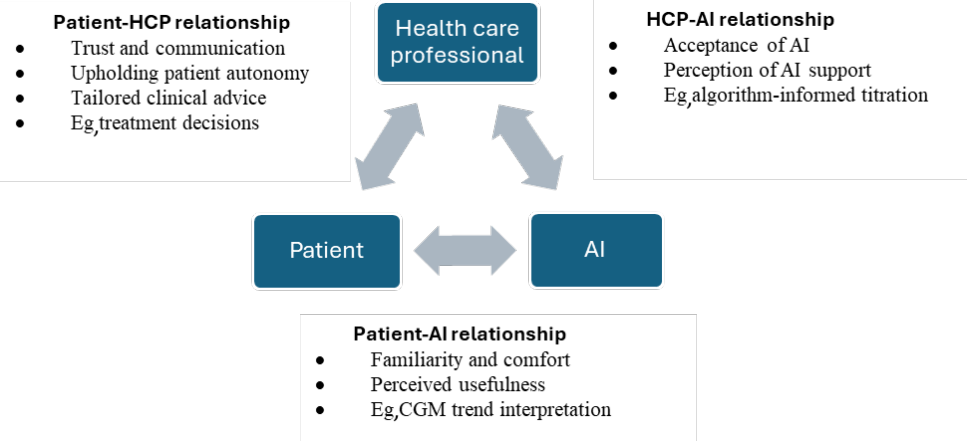
The triadic shared decision-making framework integrating artificial intelligence (AI) in diabetes care. CGM: continuous glucose monitoring; HCP: health care professional.

Our proposed triadic model expands traditional SDM by incorporating AI as an additional decision partner (Figure 1). This model is a theoretical extension of existing SDM models, incorporating AI as a third actor based on prior literature and clinical reasoning. It is not presented as an empirically validated model but as a conceptual lens that focuses on patient perceptions of 3 key relationships—patient-HCP, patient-AI, and HCP-AI—and how these shape preferences for decision support across specific diabetes self-management tasks.

*Patient-HCP relationship* refers to the patient’s perception of their HCPs’ involvement in their care and reflects trust, communication, and confidence in the HCP to make clinical decisions. As patients take on more responsibility for daily self-management, they rely on continuous support from HCPs to ensure that their decisions are aligned with long-term health goals [5,28]. Our research explores whether AI is likely to require HCPs to continue to support patients or, alternatively, whether it would allow patients to feel more autonomous and less reliant on their HCPs.

*Patient-AI relationship* refers to the patient’s familiarity and perceived usefulness of AI for self-management tasks. AI provides personalized recommendations based on real-time data, enabling patients to make informed decisions about their care [16,24,31]. Our research examines whether a higher perceived usefulness of AI can, in fact, enhance patients’ preferences for AI. We note that perceived usefulness may reflect the anticipated value of AI, which is not always based on direct experience. This differs from the patient-HCP relationship, which often reflects accumulated lived interactions.

*HCP-AI relationship* refers to the patients’ acceptance of HCPs using AI to inform care. Current research suggests that HCPs remain the primary decision-makers for tasks requiring clinical judgment and empathy, particularly for complex cases where patients rely on their expertise [21,31]. Our research explores whether patients accept HCPs using AI in their treatment and for which tasks.

AI is not treated as a monolithic tool but conceptualized along a spectrum from passive (eg, data collection) to active (eg, predictive suggestions) to actively assistive (eg, closed-loop insulin pumps). Each actor contributes to different phases of diabetes self-management, ranging from data tracking to contextualized decisions, depending on the nature of the task and the capabilities of the support system.

Bidirectional arrows between AI and patients/HCPs are used to represent perceived interactions**,** not literal reciprocity. While AI does not reciprocate relationships, it can offer feedback loops (eg, data interpretation, tailored prompts) that patients experience as responsive or personalized. Similarly, HCPs may rely on AI insights to inform care decisions. In the model, these constructs are treated as parallel relational perceptions rather than equivalent forms of experience.

### Self-Management Tasks

This study focuses on specific self-management tasks to address 2 overarching types of processes: data-driven tasks and contextualized decision-making tasks (Textbox 1).

*Data-driven tasks* include activities such as collecting health data, interpreting trends, and evaluating general treatment information. These tasks are supported by current AI technologies (eg, CGMs, mobile health applications with predictive capabilities). Patients often perform these tasks independently, with AI enabling tracking, interpretation, and visualization of data. In this sense, AI acts as an enabler of self-efficacy, often facilitating early insights or triage that may later be shared with HCPs. Contextualized decision-making tasks include clinical judgment (eg, selecting treatment plans, medication adherence) and personal reflection (eg, weighing personal pros and cons, aligning care with goals and values). We group them together to highlight a shared demand for understanding beyond data. These tasks rely on contextualized reasoning, relational trust, and emotional nuance. AI may support aspects of this domain through decision aids, reminders, or empathetic conversational agents, but cannot yet fully replicate the relational aspects of human care.

The 7 tasks selected for this study were informed by established patient-centered categories of daily diabetes management, particularly those outlined by Moser et al [32]. These tasks represent broad, recurring decision-making processes that patients commonly navigate in routine care, rather than an exhaustive list of all diabetes-related behaviors. This classification also reflects the fundamental functions of AI, which range from collecting and processing information (often referred to as big data approaches) to learning from this data through machine learning, and ultimately adapting and personalizing outputs to individual contexts [33]. Previous research highlights the value of AI-driven tools to enhance patient knowledge and tailor support to their real-time needs [3]. In this sense, the selected tasks reflect high-level domains where decision-making could plausibly be distributed between patients, HCPs, and AI systems.

Textbox 1. **Patients’ diabetes self-management strategies and tasks associated with them.**

**Data-driven tasks**

- Collecting and tracking health data (eg, blood glucose levels and medical history): routine data capture and monitoring supported by wearables and sensors.
- Interpreting diabetes data (eg, analyzing blood sugar levels and recognizing potential problems or complications that may arise): AI tools can detect patterns and provide alerts and/or predictions.
- Evaluating medical information, including pros and cons of different treatments: focuses on information retrieval and comparative decision support by identifying or summarizing research evidence (eg, guidelines) to present evidence-based options.

**Contextualized decision-making tasks**

- Personal reflection (ie, sharing thoughts and feelings about personal circumstances, goals, beliefs, and values): reflecting on personal health beliefs to weigh up personal pros and cons of treatment and lifestyle options.
- Lifestyle management (ie, managing diet, exercise, and lifestyle): integrating healthy eating, physical activity, and social and emotional support.
- Medication adherence (ie, taking medications correctly): following prescribed medication schedules and ensuring consistent use of medications. Requires a mixture of habit-forming and behavioral support.
- Seeking medical advice on how to manage diabetes/deciding on the best treatment plan: involves seeking guidance and making informed decisions when encountering unexpected health issues or irregularities.

## Methods

### Study Design

The study used a cross-sectional quantitative survey design. We followed the STROBE (Strengthening the Reporting of Observational Studies in Epidemiology) guideline for observational studies (Checklist 1) and the online survey component is reported according to the CHERRIES (Checklist for Reporting Results of Internet E-Surveys) checklist (Checklist 2).

### Ethical Considerations

Ethical approval was granted by the University of Auckland Human Participant Ethics Committee (Reference number 26326). Consent was obtained via participants clicking a button to show they had read and accepted participation in the study. No identifying information (including IP addresses) was collected. Data were stored on a secure, password-protected server at the University of Auckland. As compensation, participants could enter a draw to win 1 of 2 US $90 supermarket vouchers to acknowledge their participation.

### Recruitment

Participants were recruited via convenience sampling from New Zealand (NZ) diabetes organizations, including Diabetes NZ, Manawatu Horowhenua Tararua Diabetes Trust, North Shore Diabetes Support Group, Diabetes Wellington, and Diabetes Trust Palmerston North, as well as diabetes-focused Facebook groups and online communities. These organizations were contacted through email, telephone, or private messaging to request their assistance and consent to advertise the questionnaire to their members through their channels. Inclusion criteria were NZ residents aged 18 and older who had been diagnosed with type 1 or type 2 diabetes. To participate, individuals were not required to have prior experience with AI. Because the survey link was openly distributed through diabetes organizations and social media, the number of individuals who viewed the invitation could not be determined, and participation and completion rates could not be precisely calculated.

### Survey Design

As no existing validated instrument captured the task-specific distribution of AI versus HCP involvement in diabetes self-management, bespoke items were developed for this study. The quantitative survey was informed by existing literature and expert consultation and was designed to explore participants’ perceptions of AI and their preferences for AI versus HCP involvement across a range of self-management tasks. Because each task reflects a distinct and conceptually independent aspect of diabetes self-management, these items were treated as independent ratings rather than components of a unified scale. For this reason, no internal consistency or reliability testing was conducted. The questionnaire used a fixed item order; no randomization or adaptive questioning was applied. Participants were able to review and change their responses using a back button throughout the survey.

The survey consisted of 5 sections comprising 26 mandatory questions. The first section assessed the impact of diabetes on participants’ daily life, including physical, emotional, social, financial, as well as what strategies they currently used to manage their condition (eg, blood glucose monitoring, medication use, dietary changes, and stress management). The second section examined motivation, knowledge, and perceived control of their diabetes. Participants were asked how motivated they felt to manage their diabetes, how this motivation had changed over time, how knowledgeable they felt about their condition, and how much control they believed they had over their diabetes.

The third section assessed the importance of specific tasks and the current role of HCPs in managing these tasks. Participants rated the importance of 7 diabetes self-management tasks and the degree to which their HCP was currently involved in each. These tasks were presented with high-level descriptors. For example, “sharing your thoughts and feelings about your personal circumstances, goals, beliefs, and values” was used to represent the task we categorized as *personal reflection*.

The fourth section focused on use and perception of AI. Participants were provided with a definition of AI in the context of diabetes care:

Artificial Intelligence (AI) is increasingly being used in healthcare to help people manage their diabetes. For instance, an app could use AI to analyze a person’s diet, medication, exercise, and blood sugar levels, and then give personalized advice to help them control their blood sugar. Additionally, AI can be used in other ways, including wearable devices (eg, smart watches), continuous glucose monitoring devices (eg, Dexcom™, Freestyle Libre™), computerized insulin pumps, or diagnostic tools used by clinicians.

Participants were asked whether they currently used AI for each of the diabetes tasks (Yes/No), how useful they would find AI for those tasks, and how comfortable they were with their HCP using AI to assist in their care. The survey did not focus on specific AI technologies used.

The final section collected demographic information, including age, gender, ethnicity, education level, and region of residence.

### Data Collection

The questionnaire was piloted with 6 people (3 with type 2 diabetes) for clarity, comprehension, and wording, but did not include psychometric validation. Based on feedback, jargon and ambiguous terminology were reworded, repetitive or obscure questions were removed, and further examples of AI were given to aid comprehension. Pilot survey results were excluded from the final dataset. Qualtrics (Qualtrics) was used to create, distribute, and manage the questionnaire and data collection. The software afforded participants anonymity and allowed for convenient distribution. Data were collected from September 1 to 30, 2023.

### Statistical Analysis

While the survey included broader items on motivation and disease impact, this study reports only responses relevant to AI-supported care and SDM. Additional analyses will be reported in future publications.

Descriptive statistics (means, SDs, percentages) were used to summarize participant demographics and survey responses. For each self-management task, 4 key variables were derived:

1. Perceived HCP involvement (patient-HCP relationship)—rated on a 5-point Likert scale (1=not at all, 5=a great deal).
2. Perceived usefulness of AI (patient-AI relationship)—rated on a 5-point Likert scale (1=not at all, 5=a great deal).
3. Comfort with HCP using AI (HCP-AI relationship)—rated on a 7-point Likert scale (1=strongly disagree, 7=strongly agree).
4. Preferences for a human HCP provider—rated on a 5-point scale (1=HCP only, 3=AI and HCP equally, 5=AI only).

Task-specific preferences for AI were analyzed using both ordinal logistic regression and ordinary least squares (OLS) regression on the 5-point outcome. This dual approach is consistent with recommendations in behavioral and health research, where 5-point Likert-type variables are frequently treated as either ordered categories or approximately interval. Extensive simulation work shows that parametric analyses remain robust to violations of interval-level assumptions when scales contain 5 or more response categories [34,35]. For tasks 2‐5, where the proportional-odds assumption was satisfied, ordinal logistic regression served as the primary analysis; for tasks 1 and 6, where this assumption was violated, interpretation relied on the corresponding OLS estimates.

The patient-HCP, patient-AI, and HCP-AI relationships were entered simultaneously (enter method) rather than using stepwise selection to minimize the risk of overfitting and enhance interpretability.

Data analysis was performed using SPSS (version 29; IBM Corp), and statistical significance was set at *P*<.05. Adjusted *R*² values are reported to indicate model fit. Due to the modest sample size (N=48), findings are interpreted as exploratory and hypothesis-generating. Post hoc power calculations indicate that with 5 predictors in the regression models, this study had approximately 80% power to detect an overall model effect of about *R*²≈0.23 (Cohen *f*²≈0.30) at α=.05. Power to detect smaller effects (eg, *R*²=0.10) was low (~0.34). Accordingly, nonsignificant findings should not be interpreted as evidence of no association.

## Results

### Participant Demographics

A total of 48 participants (N=48) completed the survey. Of the 48 participants, 27 (56.3%) had been living with diabetes for more than 10 years, 38 (79.2%) were female, 27 (56.3%) were aged 26 to 45 years, and 36 (75%) identified as European. A majority had higher education qualifications (26/48, 54.2%). Participants were distributed throughout New Zealand, with the highest concentration of participants located in Auckland and Coromandel (15/48, 31.3%) (Table 1).

**Table 1.** Participant demographics (N=48).

Characteristic	Participants, n (%)
Age, y	
18-25	9 (18.8)
26-35	14 (29.2)
36-45	13 (27.1)
46-55	7 (14.6)
56-65	2 (4.2)
>65	3 (6.3)
Gender	
Female	38 (79.2)
Male	8 (16.7)
Ethnicity	
Asian	3 (6.3)
Māori	4 (8.3)
MELAA^a^	1 (2.1)
European	36 (75)
Pacific People	1 (2.1)
Other	3 (6.3)
Education	
Some secondary school	3 (6.3)
Completed secondary school	8 (16.7)
Technical certificate	10 (20.8)
Bachelors degree	19 (39.6)
Postgraduate degree	7 (14.6)
Other	1 (2.1)
Diabetes diagnosis duration	
<1 y	4 (8.3)
1‐5 y	10 (20.8)
5‐10 y	8 (16.7)
>10 y	26 (54.2)
Region	
Northland	2 (4.2)
Auckland and Coromandel	15 (31.3)
Waikato	6 (12.5)
Manawatu	4 (8.3)
Bay of Plenty	1 (2.1)
Hawkes Bay and Gisborne	1 (2.1)
Wellington	9 (18.8)
Canterbury	8 (16.7)
Otago	1 (2.1)
Southland	1 (2.1)

a Middle Eastern, Latin American, African.

### AI Use and Perception in Diabetes Management

Participants rated HCP involvement in diabetes management tasks as modest overall (mean 2.82, SD 1.23; 5-point scale; Table 2). Involvement was particularly low in tasks related to lifestyle management (mean 2.28, SD 1.19) and personal reflection (mean 2.44, SD 1.35). In contrast, participants rated AI as moderately useful (mean 3.67, SD 1.20; 5-point scale). AI was seen as particularly useful for collecting and tracking data (mean 4.23, SD 1.06) and for interpreting diabetes data (mean 4.40, SD 0.87). However, the actual use of AI across all tasks remained relatively low (mean usage rate of 31%), suggesting a gap between perceived usefulness and real-world adoption. AI use was most common for tasks involving health data tracking and interpretation, and least common for personal reflection.

Participants’ comfort with HCPs using AI was high across all tasks (mean 5.27; 7-point scale), indicating general openness to AI-assisted care, especially when used by an HCP in their care. There was a noteworthy preference for AI where the utility was already recognized (tasks 1 and 2).

**Table 2.** Mean (SD) scores of survey data related to diabetes management tasks and the use and perception of artificial intelligence (AI) in these tasks (N=48).

	Patient-HCP^a^ relationship	Patient-AI relationship		HCP-AI relationship	Patient’s relationship with HCP and AI
Tasks	HCP’s current involvement (1=Not at all; 3=Moderate; 5=A great deal)	AI usefulness (1=Not at all; 3=Moderate; 5=Extremely)	Current use of AI (0=No; 1=Yes)	Agree with HCP using AI (1=Strongly disagree; 5=Somewhat agree; 7=Strongly Agree)	Preference for 1=HCP only; 3=HCP & AI; 5=AI only^b^
Task 1: Collecting and tracking health data	3.10 (1.15)	4.23 (1.06)	0.58	5.81 (1.23)	3.48 (0.85)
Task 2: Interpreting diabetes data	3.02 (1.19)	4.40 (0.87)	0.50	5.71 (1.27)	3.25 (0.81)
Task 3: Seeking medical advice and making treatment decisions	3.06 (1.21)	3.60 (1.17)	0.25	5.30 (1.69)	2.77 (1.04)
Task 4: Lifestyle management	2.28 (1.19)	3.52 (1.37)	0.21	4.98 (1.82)	3.26 (0.85)
Task 5: Medication adherence	3.00 (1.34)	3.83 (1.2)	0.32	5.42 (1.67)	2.90 (1.04)
Task 6: Personal reflection	2.44 (1.35)	2.61 (1.44)	0.09	4.33 (1.94)	2.54 (1.17)
Task 7: Evaluating medical information	2.83 (1.19)	3.52 (1.27)	0.23	5.35 (1.54)	2.88 (0.94)
Overall mean	2.82 (1.23)	3.67 (1.20)	0.31	5.27 (1.59)	3.01 (0.96)

a HCP: health care professional.

b Preference ratings indicate participants who preferred to perform each task.

### Participant Preferences for AI Versus HCPs

**Figure 2.**
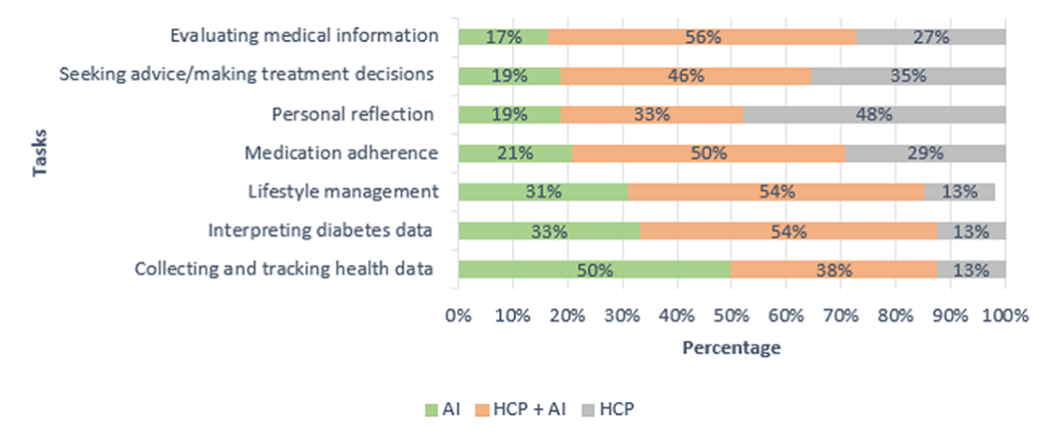
Bar graph showing the percentage of patients who preferred artificial intelligence (AI) or health care providers (HCPs) for each diabetes task.

When asked to choose between AI, HCPs, or both for completing specific tasks, participants demonstrated a clear task-specific preference pattern (Figure 2). They favored AI for data-driven tasks, such as collecting and tracking data (mean 3.48, 50%) and interpreting data (mean 3.25, 33%), while HCPs were preferred for contextualized tasks involving clinical judgment (eg, treatment decisions, mean 2.77, 35%) and personal reflection (mean 2.54, 48%).

Table 3 summarizes the results of the OLS and ordinal logistic regression models. Across both modeling approaches, with the exception of 2 effects in task 5, consistent patterns emerged. The participants’ perceived usefulness of AI (the patient-AI relationship) was a positive predictor of AI preference for four tasks: collecting/tracking health data, interpreting data, seeking medical advice/treatment decisions, and lifestyle management, with no significant effects observed for the remaining tasks. These associations suggest that individuals who perceive AI as valuable may be more inclined to accept its use across a range of diabetes self-management functions. Conversely, the patient-HCP relationship negatively predicted AI preference for treatment decision-making (both models) and medication adherence (OLS model only). These findings suggest that stronger engagement with HCPs reduces preference for AI in tasks requiring clinical judgment or behavioral support. Perceptions of the HCP-AI relationship (ie, their comfort with clinicians using AI) showed minimal influence, reaching significance only for medication adherence in the ordinal model. Gender and age did not reliably predict AI preferences. The convergence across analytical approaches strengthens confidence in the robustness of these findings despite the modest sample size.

**Table 3.** Regression results (enter method) predicting participants’ preferences for artificial intelligence (AI) versus health care professional (HCP).^a^

Predictor/ summary	Task 1: collecting and tracking	Task 2: interpreting data	Task 3: medical advice/treatment decisions	Task 4: lifestyle management	Task 5: medication adherence	Task 6: personal reflection
OLS^b^ model summary	*F*(5,42)=2.05 *P*=.09 Adj *R*^2^=0.10	*F*(5,42)=2.39 *P*=.05 Adj *R*^2^=0.13	*F*(5,41)=3.22 *P*=.015 Adj *R*^2^=0.19	*F*(5,39)=1.72 *P*=.15 Adj *R*^2^=0.08	*F*(5,41)=3.52 *P*=.01 Adj *R*^*2*^=0.21	*F*(5,40)=2.22 *P*=.07 Adj *R*^2^=0.12
Ordinal model summary	*χ*²(5)=9.19 *P*=.10 *R*^2^=0.19 PO^c^ violated	*χ*²(5)=11.71 *P*=.04 *R*^2^=0.24 PO met	*χ*²(5)=17.16 *P*=.004 *R*^2^=0.33 PO met	*χ*²(5)=8.54 *P*=.13 *R*^2^=0.19 PO met	*χ*²(5)=17.14 *P*=.004 *R*^2^=0.33 PO met	*χ*²(5)=11.30 *P*=.046 *R*^2^=0.23 PO violated
Patient-HCP	OLS: ns^d^ Ordinal: ns	OLS: ns Ordinal: ns	OLS: –.27 (.12) *P*=.03 Ordinal: –.67 (.26) *P*=.01	OLS: ns Ordinal: ns	OLS: *–.*32 (.11) *P=*.005 Ordinal: ns	OLS: ns Ordinal: ns
Patient-AI	OLS: .32 (.13) *P*=.02 Ordinal: .76 (.34) *P*=.02	OLS: .39 (.13) *P*=.005 Ordinal: 1.02 (.38) *P*=.007	OLS: .31 (.15) *P*=.04 Ordinal: .63 (.32) *P*=.049	OLS: .25 (.12) *P*=.046 Ordinal: .63 (.32) *P*=.049	OLS: ns Ordinal: ns	OLS: ns Ordinal: ns
HCP-AI	OLS: ns Ordinal: ns	OLS: ns Ordinal: ns	OLS: ns Ordinal: ns	OLS: ns Ordinal: ns	OLS: ns Ordinal: .48 (.24) *P*=.04	OLS: ns Ordinal: ns
Gender (female)	OLS: ns Ordinal: ns	OLS: ns Ordinal: ns	OLS: ns Ordinal: ns	OLS: ns Ordinal: ns	OLS: ns Ordinal: ns	OLS: ns Ordinal: ns
Age (≥36)	OLS: ns Ordinal: ns	OLS: ns Ordinal: ns	OLS: ns Ordinal: ns	OLS: ns Ordinal: ns	OLS: ns Ordinal: ns	OLS: ns Ordinal: ns

a OLS: B (SE); ordinal: B (SE); OLS significance based on *t* tests; ordinal significance based on Wald tests.

b OLS: ordinary least squares.

c PO: proportional odds assumption.

d ns: not significant.

## Discussion

### Principal Results

This study explored how patients with diabetes perceive the role of AI in their self-management, focusing on task-specific preferences and perceived relationships with HCPs and AI systems. Participants generally rated their HCPs’ involvement as moderate to low across most self-management tasks, especially for lifestyle support and personal reflection. In contrast, AI was rated as moderately useful, particularly for data-focused tasks like tracking and interpreting health data, although actual use of AI technologies remained limited. These results suggest a potential disconnect between perceived utility and real-world uptake, which may reflect accessibility, familiarity, or trust in existing AI tools.

### Comparison With Prior Work

Clear patterns emerged in how participants allocated tasks between AI and HCPs. Consistent with prior research on technology acceptance theory [36], participants who viewed AI as useful were more likely to prefer AI involvement in data-driven tasks such as data collection and tracking and interpreting health information. These domains align with established strengths of digital tools, such as CGM devices, smartwatches, and smartphone applications in managing health data. These tools are valued for their efficiency, accuracy [31,37], and the ability to provide real-time insights, excelling in areas requiring routine monitoring and pattern recognition—areas where human oversight may be more prone to errors or delays [3,17,19,21,22]. Our findings also showed that many participants perceived AI as useful, despite having limited personal experience with AI-enabled tools, highlighting a conceptual rather than experiential form of acceptance. This distinction is important, as users may associate step tracking or glucose monitoring with AI, even when the device itself lacks predictive or adaptive features. Although the study employed a broad and accessible definition of AI in the survey, AI capabilities vary widely, and the line between “monitoring” and “intelligent assistance” is often blurred. The gap between perceived usefulness and actual use may also reflect broad awareness of AI’s potential coupled with limited access to more advanced tools, leading participants to endorse AI aspirationally rather than from direct experience.

In contrast, participants preferred HCPs for tasks involving clinical judgment (particularly medication and treatment decisions) and personal reflection (eg, goal alignment). The regression analyses indicated that stronger patient-HCP relationships were associated with lower preference for AI in clinically sensitive tasks, tentatively highlighting the importance of human involvement in tasks requiring contextual judgment and personalized clinical guidance [38]. While some studies suggest a strong, ongoing preference for HCPs over AI [24,39], others highlight emerging support for a blended approach that combines the efficiency and analytical strengths of AI with the relational strengths of HCPs [17,21,40]. These mixed preferences are likely due to the still-evolving nature of AI applications and their ability to deliver contextually relevant recommendations. For instance, AI shows promise in improving the accuracy of dietary monitoring and enhancing patients’ understanding of complex medical information tailoring [3,41], but limited adoption may stem from challenges in addressing cultural and individual preferences [39]. In New Zealand, Māori and Pacific Peoples have shown a strong preference for human involvement in health care, reflecting cultural values that prioritize personal connections and face-to-face interactions in medical decision-making [21]. Importantly, our findings suggest that patients with weaker HCP relationships may view AI as a potential source of support where relational or clinical continuity is lacking. While this interpretation remains exploratory, it aligns with the broader aim of digital tools to enhance patient empowerment and self-engagement in care [38]. In cases where patients feel unsupported, AI may offer a sense of responsiveness or access. However, the diversity of AI tools in the marketplace is likely to contribute to varied levels of acceptance, reinforcing the need to assess AI preferences at a task and technology-specific level rather than assuming uniform acceptance or rejection.

An important finding in this study was the low perceived usefulness of AI for personal reflection, where participants overwhelmingly favored HCPs. Interestingly, participants also rated their HCP’s current involvement in personal reflection as below average, highlighting a broader gap in support for these more relational aspects of care. This contrasts with evidence showing that patients value opportunities to reflect on how diabetes affects their symptoms, daily lives, and treatment plans [9,42]. One possible explanation for this discrepancy is uncertainty about the relevance of personal reflection to SDM or limited prior experience with these conversations in routine care. Patients may also hesitate to express vulnerability to a non-human entity, given their reliance on HCPs for empathy, compassion, and trust to aid decision-making [43]. Given AI’s current inability to replicate these human attributes fully, there are understandable doubts about its effectiveness in offering personal support or facilitating deeper patient-HCP relationships. However, recent developments in generative AI suggest this may be changing. Studies show that AI systems can exhibit empathetic tone and rapport that, in some cases, outperform physicians in perceived bedside manner [44,45]. Chatbots have also shown promise in supporting mental health, particularly where users value guidance or nonjudgmental listening [46]. Low perceived usefulness in our study may therefore reflect a lack of access or experience with these tools rather than inherent limitations.

Although the HCP-AI variable was not a significant predictor in our regression models, this measure reflects only participants’ comfort with clinicians using AI and does not fully capture the broader concept of HCP-AI collaboration within decision-making. Taken together, these findings suggest that while AI may have the potential to support SDM, its integration into clinical workflows may not yet be perceived as necessary for effective collaboration. Previous studies support the notion that patients are generally comfortable with AI technologies being used by HCPs for screening and diagnosis, particularly when clear physician oversight is maintained [21,47]. Such supervision provides reassurance, increasing patient confidence in AI outputs when mediated by their clinicians. For HCPs, delegating routine tasks to AI could theoretically free up time for higher-value interactions with patients, enhancing personalized diabetes care [3], improving diagnostic accuracy, and reducing bias in treatment-related decisions [21]. However, the potential of AI in data collection and tracking remains underrecognized despite its demonstrated efficiency and utility in these tasks [48,49].

### Limitations

Several limitations should be considered when interpreting the findings of this study. While we propose a triadic AI-SDM model involving patients, HCPs, and AI tools, the regression analyses conducted in this study treated each relationship (patient-HCP, patient-AI, HCP-AI) as an independent predictor of AI preference. This statistical approach allowed exploration of relational perceptions individually, but did not fully capture the collaborative and interactive dynamics central to SDM. Furthermore, although the task preference measure was adapted from the Control Preferences Scale, it assessed which actor (AI, HCP, or both) participants preferred to perform each task, rather than the extent of collaboration or shared agency. As such, our findings reflect task assignment preferences rather than preferences for co-deliberation or SDM processes. Additionally, the perceptions of AI usefulness may not align with future real-world experiences, particularly since participants varied in their direct exposure to AI tools. This perception–experience gap is typical in early adoption cycles and should be evaluated longitudinally in future work.

We also did not measure or adjust for several clinical and experiential factors that could plausibly influence both perceived usefulness and preferences for AI, such as diabetes type, duration of diabetes, comorbidity burden, prior use of digital health tools (eg, CGMs, apps), or digital health literacy. Unmeasured confounding may therefore contribute to the observed associations. Future research with larger samples should incorporate these covariates and test more fully adjusted models.

Because this was a self-report survey, responses may also be subject to recall bias, social desirability bias, and variations in participants’ interpretations of key terms. Although we provided a standardized definition of “AI,” individual mental models of AI likely differed, given the rapid evolution of AI technologies and varying levels of familiarity among participants. The 5-point preference scale included a midpoint (“both equally”), but we cannot determine how consistently respondents interpreted this option. Several constructs were measured using single-item indicators, which can introduce measurement noise even though this approach reduced respondent burden. Responses may also have been influenced by the online survey format, including differences in device type or environmental distractions.

The modest sample size further limits statistical power and increases the risk of type II error, meaning that small to moderate associations may have been missed. In addition, estimates from multivariable models in small samples can be unstable (ie, sensitive to small changes in the data), so coefficients and standard errors should be interpreted cautiously. In line with the post-hoc calculations reported above, the study was best powered to detect moderate to large overall effects.

Over half of our population had higher qualifications, and 75% were between 18 and 45 years old, with 66.7% living in larger cities in New Zealand, which can be attributed to the location of diabetes organizations that promoted the survey. This recruitment approach may have disproportionately attracted individuals who are more digitally engaged or open to technology, potentially inflating the perceived usefulness of AI relative to the broader diabetes population. Māori were underrepresented compared to national averages [50] as were older adults, who often have different cultural expectations and relational preferences in health care. Thus, our findings should be interpreted as exploratory and reflective of a digitally engaged subset rather than the general New Zealand diabetes population. Additionally, the absence of qualitative data limits our ability to fully understand the potential fears, mistrust, or contextual factors influencing participants’ preferences and attitudes toward AI and HCP collaboration. Data-wise, the study’s adjusted *R*² values ranged from 0.08 to 0.21, indicating that our model explains only a small proportion of the variance in AI-related preferences. This suggests that other unexplored factors, such as familiarity with technology or past experiences with AI, likely play a role in shaping preferences. These modest values suggest that AI preferences are influenced by numerous additional factors not accounted for in the current models, and the observed associations should therefore be interpreted as limited and exploratory.

### Implications for Health Care

Integrating AI into diabetes care offers a promising opportunity to address the challenges in primary care settings, including time constraints, limited staffing, and the growing complexity of chronic disease management [24,51]. Our findings suggest that participants in this sample appeared more receptive to AI for data-driven, routine tasks, such as collecting, tracking, and interpreting information, where automation could relieve HCP workload and increase system efficiency. This aligns with our regression results, which showed strong associations between perceived usefulness of AI and preferences for AI involvement in these tasks. In contrast, participants preferred HCP involvement in treatment decision-making, medication adherence, and personal reflection, emphasizing the importance of human relationships and relational trust in these domains.

For HCPs considering the integration of AI into practice, several actionable recommendations emerge:

- Prioritize AI for backend data analysis, pattern recognition, and patient self-monitoring tools, where patient preference for automation is stronger. Use AI outputs to inform, but not replace, clinical conversations.
- HCPs should clearly explain to patients when and how AI tools are being used, especially in emotionally sensitive or decision-making contexts, to avoid undermining trust.
- Recognize that some patients place a high value on personal, face-to-face care. AI integration strategies should respect and adapt to these cultural expectations, ensuring that relational care is never sacrificed for technological efficiency [52].

It is also important to recognize that AI tools are not without risk. Black-box AI systems lacking transparency erode patient trust and complicate informed decision-making [28,53,54]. While human-in-the-loop models offer oversight, they are not infallible and may still perpetuate bias or introduce complacency. Additionally, the deployment of AI raises broader ethical considerations related to patient safety (eg, erroneous recommendations or automation bias), data privacy (eg, extensive data collection or unclear data-sharing pathways), and accountability for AI-supported decisions, all of which must be addressed to ensure safe integration into clinical practice. Framing AI purely as a supportive tool without critical evaluation overlooks these challenges. Instead, AI should be incorporated through task-specific validation, transparent design, ethical oversight, and continuous evaluation of patient experience.

Given the modest sample size and exploratory nature of this study, further research is needed to gain a deeper understanding of how different patient populations perceive and experience AI in care. Qualitative studies are especially necessary to explore patients’ emotional safety, cultural expectations, and boundaries around AI use in relational care. Future work should also assess how specific AI tools impact trust, decision-making, and patient-HCP dynamics across diverse populations.

### Conclusions

This study examined how people with diabetes perceive the role of AI in key self-management tasks and how their preferences compare with the role of HCPs. Across the sample, AI was viewed as most useful for data-driven tasks such as collecting, tracking, and interpreting health data, where its accuracy and efficiency excel. However, tasks requiring contextual judgment, emotional support, or personal reflection remain firmly within the domain of human providers.

While these findings offer early insight into how patients may distribute roles between AI and clinicians, they should not be interpreted as empirical validation of the proposed triadic AI–SDM framework. Instead, they highlight how patient preferences are shaped by the interplay of perceived usefulness of AI and the strength of existing patient-HCP relationships. Participants with stronger relational trust in their clinicians were less willing to allocate clinically sensitive tasks to AI, highlighting the enduring importance of human judgment and contextual understanding in SDM. Although the HCP-AI variable was not a significant predictor, this reflects the narrow focus of the survey item rather than the broader relevance of HCP-AI collaboration. Taken together, these results suggest that AI may play a complementary role in SDM, but its integration must reinforce, rather than displace, the relational foundations of person-centered care.

Future work should assess how preferences evolve over time as AI tools become more capable, regulated, and socially familiar. Longitudinal designs (eg, repeated surveys or cohort follow-up) could determine whether increasing exposure to AI-enabled diabetes technologies, improvements in explainability and safety, and shifting norms about AI-mediated care change the balance patients prefer between AI and HCP involvement, particularly for higher-stakes or more relational tasks.

Given the exploratory design, modest sample, and digitally engaged participant group, these findings should be interpreted cautiously. Nonetheless, they provide foundational insights to guide task-specific AI integration into diabetes care. Future research should examine how AI preferences vary among older adults, Indigenous communities, and rural populations and should prioritize qualitative inquiry to explore emotional safety, trust boundaries, and culturally appropriate models of AI in chronic disease management. A broader exploration of technology types, relationship dynamics, and collaborative decision-making will be essential as AI becomes increasingly embedded in chronic care.

## Supplementary material

10.2196/83030 Checklist 1 STROBE checklist.

10.2196/83030 Checklist 2 CHERRIES checklist.

## Abbreviations

AI: artificial intelligence
CGM: continuous glucose monitor
CHERRIES: Checklist for Reporting Results of Internet E-Surveys
HCP: health care professional
SDM: shared decision-making
STROBE: Strengthening the Reporting of Observational Studies in Epidemiology
