# Supplementary material for: Patient Perceptions of Artificial Intelligence in Diabetes Self-Management: Cross-Sectional Survey Study
Source: JMIR Form Res. 2026 Mar 16;10:e83030. doi: 10.2196/83030 (PMC12991192; doi:10.2196/83030)
Supplement: Checklist 1 [file formative-v10-e83030-s001.pdf]

The STROBE reporting checklist

For checking that observational epidemiology research articles can be understood and used by everyone

Note

If you have not used a reporting guideline before, read about how and why to use them and check whether STROBE is the most applicable reporting guideline for your work.

Reporting guidelines are most useful when used early in research. When writing a manuscript or application, consider using the Full Guidance where you'll see explanations and examples for each item.

After writing, demonstrate adherence by completing this checklist:

1. Specify where each item is described (see Note 1).
2. Cite this checklist (See Note 2).
3. Include your completed checklist as a supplement when submitting to a journal so that future readers can use it to find information.

	Item Description	Location (or reason for not reporting)
**Title and abstract**		
1a. Indicate the study's design	Indicate the study's design with a commonly used term in the title or the abstract.	Title
1b. Abstract	Provide in the abstract an informative and balanced summary of what was done and what was found.	Abstract
**Introduction**		
2. Background / rationale	Explain the scientific background and rationale for the investigation being reported.	Introduction
3. Objectives	State specific objectives, including any prespecified hypotheses.	Last paragraph of the introduction
**Methods**		
4. Study design	Present key elements of study design early in the paper.	First paragraph - methods
5. Setting	Describe the setting, locations, and relevant dates, including periods of recruitment, exposure, follow-up, and data collection.	Methods - Recruitment
6a. Eligibility criteria	**Cohort study:** Give the eligibility criteria, and the sources and methods of selection of participants. Describe methods of follow-up. **Case-control study:** Give the eligibility criteria, and the sources and	Methods - Recruitment

	methods of case ascertainment and control selection. Give the rationale for the choice of cases and controls. **Cross-sectional study:** Give the eligibility criteria, and the sources and methods of selection of participants.	
6b. Matching criteria	**Cohort study:** For matched studies, give matching criteria and number of exposed and unexposed. **Case-control study:** For matched studies, give matching criteria and the number of controls per case.	N/A
7. Variables	Clearly define all outcomes, exposures, predictors, potential confounders, and effect modifiers. Give diagnostic criteria, if applicable.	Methods – survey design
8. Data sources / measurement	For each variable of interest give sources of data and details of methods of assessment (measurement). Describe comparability of assessment methods if there is more than one group.	Methods – survey design
9. Bias	Describe any efforts to address potential sources of bias.	Methods – recruitment<br>Discussion - limitations
10. Study size	Explain how the study size was arrived at.	No formal sample size calculation was conducted. The study used convenience sampling, and we aimed to recruit as many eligible participants as possible during the data collection period (1–30 September 2023). This approach was deemed appropriate given the exploratory nature of the research
11. Quantitative variables	Explain how quantitative variables were handled in the analyses. If applicable, describe which groupings were chosen, and why.	Methods – statistical analysis
12a. Statistical methods	Describe all statistical methods, including those used to control for confounding.	Methods – statistical analysis
12b. Statistical methods – subgroups and interactions	Describe any methods used to examine subgroups and interactions.	N/A
12c. Statistical methods – missing data	Explain how missing data were addressed.	Participants with incomplete survey responses were excluded from the final dataset. Only fully completed surveys were included in the analysis.

12di. Statistical methods – loss to follow-up	**Cohort study:** If applicable, describe how loss to follow-up was addressed.	N/A
12dii. Statistical methods – matching cases and controls	**Case-control study:** If applicable, explain how matching of cases and controls was addressed.	N/A
12diii. Statistical methods – sampling strategy	**Cross-sectional study:** If applicable, describe analytical methods taking account of sampling strategy.	N/A
12e. Statistical methods – sensitivity analyses	Describe any sensitivity analyses.	N/A
**Results**		
13a. Participant numbers	Report the numbers of individuals at each stage of the study—e.g., numbers potentially eligible, examined for eligibility, confirmed eligible, included in the study, completing follow-up, and analysed; Consider use of a flow diagram.	Results – participant demographics
13b. Participants – non-participation	Give reasons for non-participation at each stage.	N/A
13c. Participants – flow diagram	Consider use of a flow diagram.	A flow diagram was not included as the study involved a single-stage online survey and only fully completed surveys (N=48) were included in the final analysis.
14a. Descriptive data – participant characteristics	Give characteristics of study participants (e.g., demographic, clinical, social) and information on exposures and potential confounders. Present the information in a table.	Results – participant demographics<br>Table 2
14b. Descriptive data – missing data	Indicate the number of participants with missing data for each variable of interest.	No missing data were present for variables of interest, as only fully completed surveys were included in the final analysis
14c. Descriptive data – follow-up time	**Cohort study:** Summarise follow-up time—e.g., average and total amount.	N/A
15. Outcome data	**Cohort study:** Report numbers of outcome events or summary measures over time. **Case-control study:** Report numbers in each exposure category, or summary measures of exposure. **Cross-sectional study:** Report numbers of outcome events or summary measures.	Results - Table 3 and 4, Figure 2

16a. Main results	Give unadjusted estimates and, if applicable, confounder-adjusted estimates and their precision (e.g., 95% confidence intervals). Make clear which confounders were adjusted for and why they were included.	Results - Table 4<br>No confounder-adjusted models were included, as the analysis was exploratory and demographic variables (age, gender) were not retained in the final models if not statistically significant
16b. Main results – category boundaries	Report category boundaries when continuous variables were categorised.	All continuous demographic variables that were categorised were reported with explicit category boundaries in Table 2.
16c. Main results – risk	If relevant, consider translating estimates of relative risk into absolute risk for a meaningful time period.	N/A
17. Other analyses	Report other analyses done—e.g., analyses of subgroups and interactions, and sensitivity analyses.	N/A
**Discussion**		
18. Key results	Summarise key results with reference to study objectives.	Discussion – principal results
19. Limitations	Discuss limitations of the study, taking into account sources of potential bias or imprecision. Discuss both direction and magnitude of any potential bias.	Discussion – limitations
20. Interpretation	Give a cautious overall interpretation considering objectives, limitations, multiplicity of analyses, results from similar studies, and other relevant evidence.	Discussion - Comparison with prior work
21. Generalisability	Discuss the generalisability (external validity) of the study results.	Discussion – limitations
**Other information**		
22. Funding	Give the source of funding and the role of the funders for the present study and, if applicable, for the original study on which the present article is based.	Funding is

1 How to specify where content is

Tell the reader where they can find information. E.g.,

- Results; paragraph 2
- Methods, Participants; paragraphs 1 & 2.
- Table 3
- Supplement B, para. 4

If you have chosen not to describe an item, explain why. You can do this in the checklist, or as a note below it.

You can describe items in the article body, or in tables, figures, or supplementary materials, and should prioritize items you feel are most important to your intended audience. The order of items in your manuscript does not need to match the order of items in this checklist. You can decide how best to structure your work.

2 How to cite

Describe how you used STROBE at the end of your Methods section, referencing the resources you used e.g.,

> 'We used the STROBE reporting guideline(1) to draft this manuscript, and the STROBE reporting checklist(2) when editing, included in supplement A'

If you use a reporting checklist, remember to include it as a supplement when publishing so that readers can easily find information and see how you have interpreted the guidance.

1. Elm E von, Altman DG, Egger M, Pocock SJ, Gøtzsche PC, Vandenbroucke JP, et al. The strengthening the reporting of observational studies in epidemiology (STROBE) statement: Guidelines for reporting observational studies. Annals of Internal Medicine [Internet]. 2007 Oct;147(8):573–7. Available from: https://www.acpjournals.org/doi/10.7326/0003-4819-147-8-200710160-00010

2. Elm E von, Altman DG, Egger M, Pocock SJ, Gøtzsche PC, Vandenbroucke JP, et al. The STROBE reporting checklist. In: Harwood J, Albury C, Beyer J de, Schlüssel M, Collins G, editors. The EQUATOR network reporting guideline platform [Internet]. The UK EQUATOR Centre; 2025. Available from: https:/resources.equator-network.org/reporting-guidelines/strobe/strobe-checklist.docx
