# Supplementary material for: Patient Perceptions of Artificial Intelligence in Diabetes Self-Management: Cross-Sectional Survey Study
Source: JMIR Form Res. 2026 Mar 16;10:e83030. doi: 10.2196/83030 (PMC12991192; doi:10.2196/83030)
Supplement: Checklist 2 [file formative-v10-e83030-s002.pdf]

Checklist for Reporting Results of Internet E-Surveys (CHERRIES)

	**Checklist for Reporting Results of Internet E-Surveys (CHERRIES)**	
Item Category	***Checklist Item***	***Explanation***
**Design**		
	Describe survey design	Cross-sectional online survey of adults with diabetes in New Zealand. Convenience sampling via diabetes advocacy groups and social media.
**IRB (Institutional Review Board) approval and informed consent process**		
	IRB approval	Approved by the University of Auckland Human Participants Ethics Committee
	Informed consent	Participants were shown study information prior to starting and provided electronic consent. The Participant Information Sheet included the length of time of the survey, how data were stored and for how long, who the investigator was, and the purpose of the study
	Data protection	Stored data is anonymously saved on a University-protected drive.
**Development and pre-testing**		
	Development and testing	Survey was developed from literature and expert review; pilot-tested with 6 individuals for clarity/usability. No reliability testing (and this is explained).
**Recruitment process and description of the sample having access to the questionnaire**		
	Open survey versus closed survey	Open survey—any adult with diabetes could participate through public links.
	Contact mode	Participants recruited via diabetes organisations' mailing lists, social media, and advocacy groups. Initial contact was online.

	**Checklist for Reporting Results of Internet E-Surveys (CHERRIES)**	
Item Category	***Checklist Item***	***Explanation***
	Advertising the survey	Recruitment information posted online by diabetes organisations.
**Survey administration**		
	Web/E-mail	Web-based survey via Qualtrics. Data captured automatically.
	Context	Survey not hosted on a website; accessed via shared recruitment links. Sample skew explained (digitally engaged users).
	Mandatory/voluntary	Voluntary survey
	Incentives	Small incentive offered (chance to enter into draw to win one of two $150 vouchers). Offer made to provide the survey results)
	Time/Date	Data collection period stated
	Randomization of items or questionnaires	Not applied
	Adaptive questioning	Not applied
	Number of Items	Five sections covering 26 questions
	Number of screens (pages)	
	Completeness check	Completeness was checked after the questionnaire had been submitted. There were no mandatory questions enforced.
	Review step	Respondents were able to review and change their answers through a Back button.
**Response rates**		
	Unique site visitor	N/A
	View rate (Ratio of unique survey visitors/unique site visitors)	N/A
	Participation rate (Ratio of unique visitors who agreed to participate/unique first survey page visitors)	Denominator unknown due to open recruitment

	**Checklist for Reporting Results of Internet E-Surveys (CHERRIES)**	
Item Category	***Checklist Item***	***Explanation***
	Completion rate (Ratio of users who finished the survey/users who agreed to participate)	48 completeness rate. Denominator unknown due to open recruitment.
**Preventing multiple entries from the same individual**		
	Cookies used	Not enabled
	IP check	IP duplicate detection was not enabled
	Log file analysis	Not used
	Registration	Not a closed survey
**Analysis**		
	Handling of incomplete questionnaires	Only complete questionnaires included in analysis.
	Questionnaires submitted with an atypical timestamp	Not applied
	Statistical correction	Not applied.
